# Encapsulation of pancreatic islet with HMGB1 fragment for attenuating inflammation

**DOI:** 10.1186/s40824-015-0042-2

**Published:** 2015-10-26

**Authors:** Eun Hee Jo, Yong Hwa Hwang, Dong Yun Lee

**Affiliations:** Department of Bioengineering, College of Engineering, Hanyang University, Seoul, 133-791 Republic of Korea; BK21 PLUS Future Biopharmaceutical Human Resources Training and Research Team, Hanyang University, Seoul, 133-791 Republic of Korea

**Keywords:** Alginate bead, Encapsulation, High mobility group box 1 (HMGB1), HMGB1 A box, Pancreatic islet transplantation

## Abstract

**Background:**

Pancreatic islet encapsulation is one way to address the disadvantages of islet transplantation. Not only does encapsulation involve bidirectional diffusion of nutrients, oxygen, and glucose, but also it protects the graft from the recipient’s immune reaction. The high mobility group box 1 (HMGB1), one of higher expression proteins in islet, can be secreted from transplanted islets and induce the inflammation. Therefore, the regulation of HMGB1-mediated inflammation is very important for successful islet transplantation. In this study, we used the HMGB1 A box, an antagonist of HMGB1 receptor in the immune cells, in the encapsulation of isolated islets as a new strategy.

**Result:**

For co-encapsulation of HMGB1 A box protein with islets, we evaluated the distribution of alginate bead diameter. The average diameter of empty alginate bead was similar to that of alginate bead with islets. When different concentrations of HMGB1 A box protein was co-encapsulated with islets, it did not affect the viability and insulin secretion function of the islets. When the alginate beads with islets plus HMGB1 A box protein were cultured with macrophage, the amount of TNF-α secreted from the macrophages was significantly attenuated when compared to cultivation of unencapsulated islets or encapsulated islets. When the alginate beads with islets plus HMGB1 A box protein were intraperitoneally xenotransplanted into the diabetic mice, the survival rate of the islets was strongly improved with 2-fold.

**Conclusion:**

Collectively, these results suggested that the encapsulation of HMGB1 A box protein might offer a protective effect in islet transplantation.

## Background

Type 1 Diabetes Mellitus (T1DM) is chronic disease that results from the autoreactive T lymphocyte-mediated destruction of beta cells in the pancreatic islets of Langerhans [1]. This autoimmune cell destruction against islets leads to a deficiency of insulin hormone in the body. To cure T1DM, insulin protein can be administered via injection or insulin pump, which are not an ultimate strategy due to their limited action. Alternatively, pancreatic islet transplantation has been recognized as a promising treatment approach for T1DM because transplanted islets can dynamically and directly control the blood glucose levels of diabetic patients [2]. However, it also has limitations, such as immune rejection reactions leading to its failure [1, 3]. In clinic, therefore, administration of various kinds of immunosuppressive medications has been used to prevent islet graft rejection; however, these medications can cause several adverse effects such as hypertension and renal failure in the patients during long-term administration [4, 5].

There have been many prior attempts to overcome the disadvantages islet transplantation, including especially pancreatic islet encapsulation with alginate hydrogel. This islet encapsulation remedy may remove the need for immunosuppressive drugs because the alginate bead could inhibit the recognition of host’s immune cells through ‘immune-isolation’, thereby reducing the inflammation [6, 7]. Also, alginate bead can allow the bidirectional diffusion of nutrients, oxygen, and glucose to the islets [8]. However, the encapsulated islets are under the condition of hypoxia at the center of a large bead [9–11]. The reason is the diffusion distance of oxygen and nutrients into the large-sized alginate bead (diameter: 500 ~ 1,000 μm). The islet cells is under the hypoxia-induced cell death, thereby releasing intracellular molecules [11, 12]. One of the releasing intracellular molecules is high-mobility group box 1 (HMGB1) protein. It is known that this HMGB1 is a non-histone protein involved in the regulation of transcription [13]. It has three domains, called the A box, B box, and C terminus tail. The HMGB1 A box has an anti-inflammatory activity, whereas HMGB1 B box has an inflammatory activity [14, 15]. Like a cytokine, it can be released from activated immune cells including macrophages, monocytes and dendritic cells, which can eventually mediate infection, injury and inflammation [13]. Interestingly, it was reported that pancreatic islets expressed predominantly the HMGB1 protein [16]. Therefore, if islets were encapsulated in the alginate bead, they could highly release the HMGB1 protein during hypoxia-induced cell death. Recently, several literature suggested that the release of HMGB1 was strongly correlated to early loss in islet transplantation [16–18]. Based on these finding, it has been required to develop a strategy for inhibition of the HMGB1 activity.

**Scheme 1:**
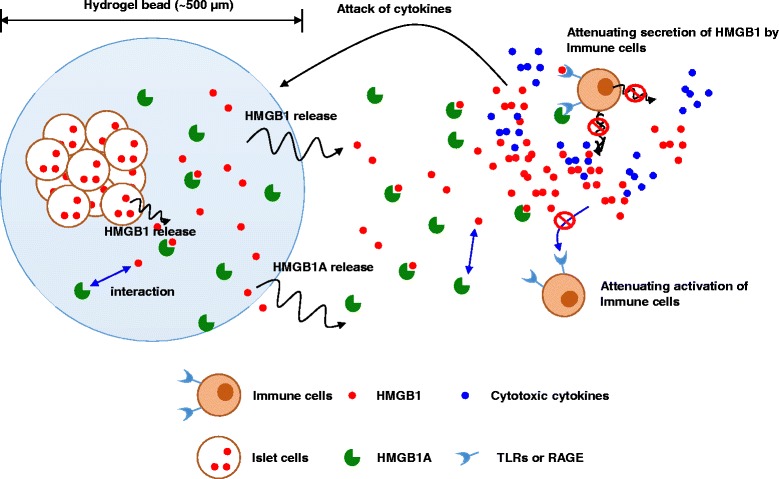
The pancreatic islet and HMGB1 A box protein were co-encapsulated in the alginate bead. The encapsulated HMGB1 A box protein may protect islets from HMGB1-mediated inflammation via competitive inhibition against TLR and/or RAGE on immune cells. By this action, this co-encapsulation of HMGB1 A box protein could reduce early graft loss in islet transplantation

To this end, here we introduced co-encapsulation of islets with HMGB1 A box fragment. We assumed that the HMGB1 A box fragment could inhibit HMGB1-mediated inflammation via competitive inhibition on the Toll-like receptors (TLRs) and the receptor for advanced glycation end products (RAGE) onto the immune cells (Scheme 1). Here we tested whether co-encapsulation of HMGB1 A box could inhibit the inflammation by using *in vitro* cultivation with macrophage and *in vivo* transplantation in diabetic animal model.

## Methods

### Islet isolation and culture

Seven-week old outbred Sprague–Dawley (SD) male rats and inbred BALB/c male mice were purchased from the Nara-Bio Company (Seoul, Republic of Korea). The animals were raised in ventilated cages under specific pathogen-free conditions at our institution. All animal procedures were approved by Hanyang University through the Institutional Animal (IACUC/ HY-IACUC-13–030A). The islets of Langerhans were isolated from the outbred SD rats using collagenase P (1 mg/mL; Roche, Basel, Switzerland). Collagenase P was injected into the pancreas through a bile duct. The pancreas organ was then harvested and digested at 37 °C for 15 min. During the separation and purification stages, the islets were washed with Medium-199 (Sigma, St. Louis, MO, USA). Finally, the isolated islets were cultured in RPMI-1640 (Invitrogen, Carlsbad, CA, USA) containing 10 % fetal bovine serum (FBS; Sigma) and 1 % penicillin-streptomycin solution (Gibco, Carlsbad, CA, USA) for 2 days.

### Preparation of HMGB1 A box protein

A bacterial host strain of *E. coli* BL21(λDE3) was used to express the vector of rHMGB1A-His_6_. The pET21a-rHMGB1A-His6 was cultured overnight in 2 ml LB medium supplemented with 50 μg/ml ampicillin in a shaking incubator at 37 °C. Bacteria were then transferred to the LB medium and cultured in the same conditions. The expression of rHMGB1A-His_6_ was induced using isopropyl-β-d-thiogalactopyranoside (IPTG, Sigma) for 6 h in culture at 25 °C. The bacteria were collected and suspended in 50 mM of NaH_2_PO_4_ (pH 8.0) containing 300 mM NaCl, 10 mM imidazole and 1 mM phenylmethylsulfonylfluoride (PMSF). The lysates were elicited using centrifugation at 10,000 × *g* for 30 min at 4 °C. To purify the rHMGB1A-His6 fusion protein in the lysates, nickel-chelate affinity chromatography (Probond resin, Invitrogen) was used. Unbound proteins were passed through the equilibration buffer, and the bound rHMGB1A-His_6_ peptides were eluted using a step-gradient of imidazole. The eluted fractions were assayed using the BCA assay kit (Pierce, Rockford, IL, USA). The fraction of purified protein was pooled, dialyzed with phosphate buffered saline (PBS) and stored at −80 °C until use.

### Generation of alginate bead

Encapsulator Biotech (EncapBioSystems Inc., Greifensee, Switzerland) was used to prepare alginate beads. Briefly, 0.15 g of alginic acid (Sigma) was dissolved in 10 ml of distilled water. The 1.5 % alginate solution was injected 10 ml syringe and then installed Encapsulator biotech. The Alginate-based bead generator conditions included a frequency of 1700 Hz, electrode 1100 V, stirrer 5 rpm, pump 33.8 ml/min and nozzle diameter of 300 μm. Calcium chloride solution (3 %) was also used for gelation of alginate beads. The alginate beads were 3-times washed with Dulbeco’s modified eagle’s medium (DMEM; GenDEPOT, Barker, TX, USA). The size distribution of alginate bead was calculated to evaluate the effect of islets and HMGB1 A box protein to the formation of alginate bead.

### Toxicity of HMGB1 A box to isolated islets

Before encapsulation, pancreatic islets were directly exposed to each concentration (0, 2.5, 5, and 10 μM) of HMGB1 A box protein and cultured at 37 °C for 1 or 4 days. After the incubation period, the viability of the islets was measured using a CCK-8 assay kit (Dojindo Laboratories, Tokyo, Japan). On the other hand, islets were encapsulated with HMGB1 A box protein-containing alginate beads using the Encapsulator, and were cultured in DMEM medium for 1 or 4 days. The viability of the encapsulated islet was measured with the CCK-8 assay.

### Glucose responsiveness of islet encapsulated with HMGB1 A box protein

The glucose-stimulated insulin secretion (GSIS) assay was performed to evaluate the function of islets. The encapsulated islet was incubated in Krebs Ringer buffered HEPES (pH 7.4) with 0.1 % (w/v) bovine serum albumin containing 2.8 mM low glucose at 37 °C for 1 h. After this pre-incubation, the encapsulated islets were incubated at 37 °C for 2 h in 2.8 mM and 20.2 mM high glucose solution, subsequently. The amount of insulin protein secreted by the islets in the low- and high- glucose solution was measured using a rat Insulin enzyme-linked immunosorbent assay kit (ELISA; Alpco Diagnostics, NH, USA). Finally, the measured insulin content was divided by the DNA content measured by a DNA Assay kit (Invitrogen) for the compensation of size variation of islets.

### Co-culture of activated macrophages with islet-containing alginate bead

The alginate beads with islets and HMGB1 A box protein were cultured with Raw 264.7 macrophage cells in order to assess the anti-inflammatory effect of HMGB1 A box protein. To activate the Raw 264.7 cells, they were treated with 500 ng/ml HMGB1 protein for 3 days. After activation, islet-containing alginate beads were co-cultured for three days. To evaluate the effect of HMGB1 A box protein, the amount of TNF-α released from the activated macrophage was measured by using ELISA (KOMA, Seoul, Republic of Korea).

### Xenotransplantation of alginate bead with islets and HMGB1 A box protein

To evaluate whether the HMGB1 A box protein could regulate the inflammation *in vivo*, the alginate beads with islets and HMGB1 A box protein were xenotransplanted into the peritoneum of streptozotocin-induced diabetic BALB/c mice (1000 islets/mice). After xenotransplantation, the blood glucose levels of the recipients were daily measured.

### Statistical analysis

All values are represented as means ± S.E.M (Standard error of the mean). The error bars represent the S.E.M. Comparisons were performed with ANOVA one-way tests (sigma Plot, Systat Software, Inc., San Jose, CA, USA). *P*-values < 0.05 were considered statistically significant.

## Results and discussion

### Characterization of alginate bead

**Fig. 1:**
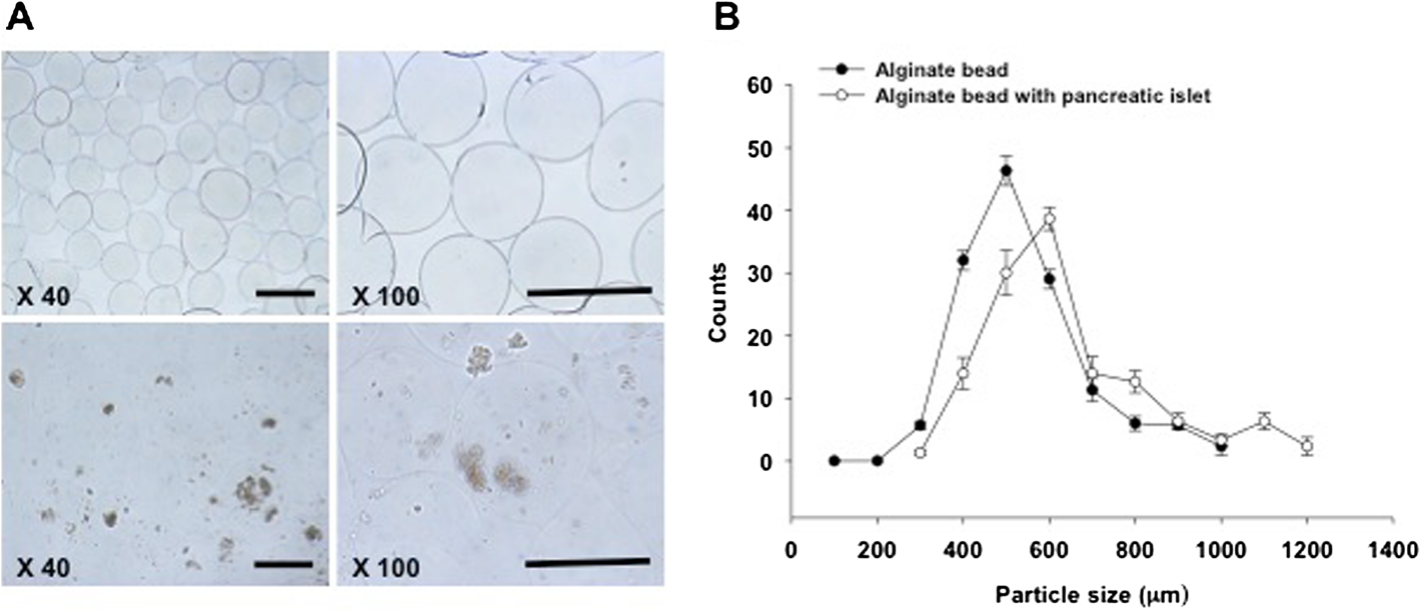
Characterization of alginate hydrogel bead. **a** Morphology of the alginate bead (upper panel) and the alginate bead with the pancreatic islet (under panel). Scale bar = 500 μm. **b** Size distribution of the alginate bead and alginate bead with pancreatic islets

After generation of alginate beads, we confirmed the morphology and size of the alginate bead with islets. The empty alginate beads and islet-containing alginate beads were uniform in circular shape (Fig. 1a). The average size of the empty alginate beads and islet-containing alginate beads was about 500 μm and 600 μm, respectively. 4.6 × 10 [4] beads per 1 ml of alginate solution were generated. The size range of the empty beads was between 300 μm and 1000 μm. The polydistribution index (PDI) of the alginate-based bead was ± 1.07, indicating that the beads were relatively uniform (Fig. 1b). On the other hand, in the case of islet-containing alginate solution, 4.4 × 10 [4] beads per 1 ml of alginate solution were generated. These ranged between 300 μm and 1200 μm in size. The islet-containing alginate beads were also uniform with a PDI of ±1.08. Therefore, based on the findings, the condition of alginate beads using Encapsulator Biotech instrument was acceptable for islet encapsulation.

### Toxicity evaluation of HMGB1 A box to isolated islets

**Fig. 2:**
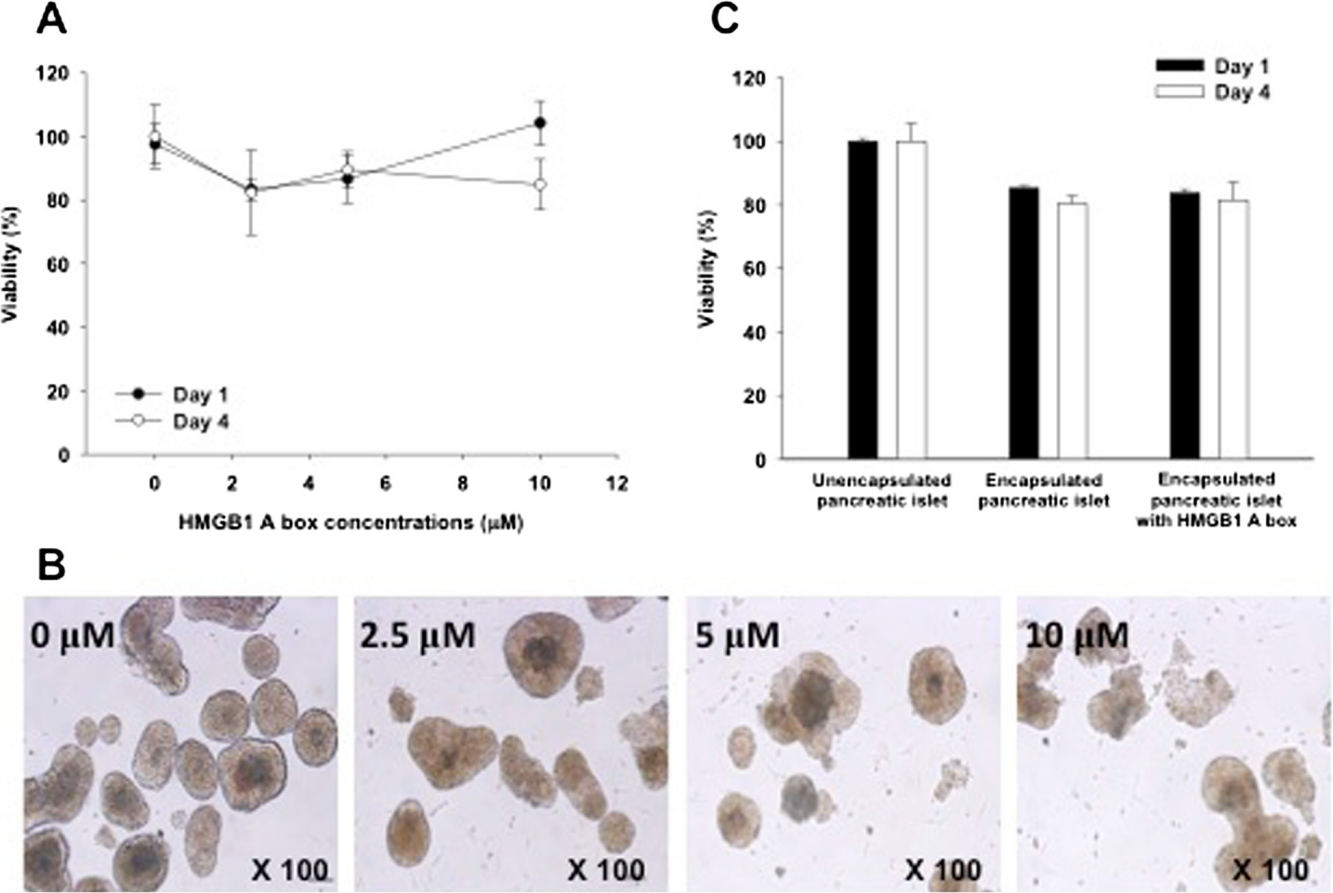
Cytotoxicity of HMGB1 A box protein to islets. **a** Effect of the HMGB1 A box (0, 2.5, 5, and 10 μM) on pancreatic islet viability for 4 days treatment. The black dot indicates the HMGB1 A box treated islet viability at day 1, and the white dot at day 4. **b** Morphology images of islets treated with HMGB1 A box protein for 4 days. **c** Viability of islets after co-encapsulation with HMGB1 A box protein. The black bar indicates the islet viability at day 1 and the white bar at day 4

To evaluate whether HMGB1 A box protein was toxic to islets, the viability of islets was measured during cultivation with different concentration of HMGB1 A box protein for 4 days (Fig. 2a). After 1-day incubation with 2.5, 5, and 10 μM of HMGB1 A box protein, the viability of islets was 83.3 ± 3.4, 86.6 ± 7.6, and 104.3 ± 6.9 %, respectively. After 4-day incubation, the viability of islets was 82.3 ± 13.3, 89.5 ± 5.9, and 84.9 ± 8.0 %, respectively. There were no statistical differences in viability between the groups. Moreover, the morphologies of islets treated with HMGB1 A box protein were not significantly different from that of untreated control islets (0 μM) after four days (Fig. 2b). From these results, we concluded that HMGB1 A box protein did not affect islet viability. The reason was that HMGB1 A box protein The HMGB1 A box concentration in alginate co-encapsulation with pancreatic islets was optimized at 10 μM.

Next, we carried out the islet encapsulation with alginate hydrogel to evaluate the viability of encapsulated islets in the presence of HMGB1 A box protein (Fig. 2c). The viability of the encapsulated islets without or with the HMGB1 A box protein after one day was 85.3 ± 0.7 and 83.8 ± 1.2 %, respectively. After 4 days, their viability without or with the HMGB1 A box was 80.4 ± 2.2 and 81.5 ± 5.5 %, respectively. There were no significant differences between the groups. Therefore, we found that HMGB1 A box protein did not affect the viability of islets in the alginate bead. However, the viability of both groups was slightly reduced when compared with unencapsulated control islets. The large bead size affects islet cell viability because it is more difficult for nutrients and oxygen to diffuse across a larger bead than it is for them to diffuse across a smaller bead. Therefore, larger beads are more likely to induce hypoxia than are smaller ones.

### Glucose responsiveness of islet encapsulated with HMGB1 A box protein

**Fig. 3:**
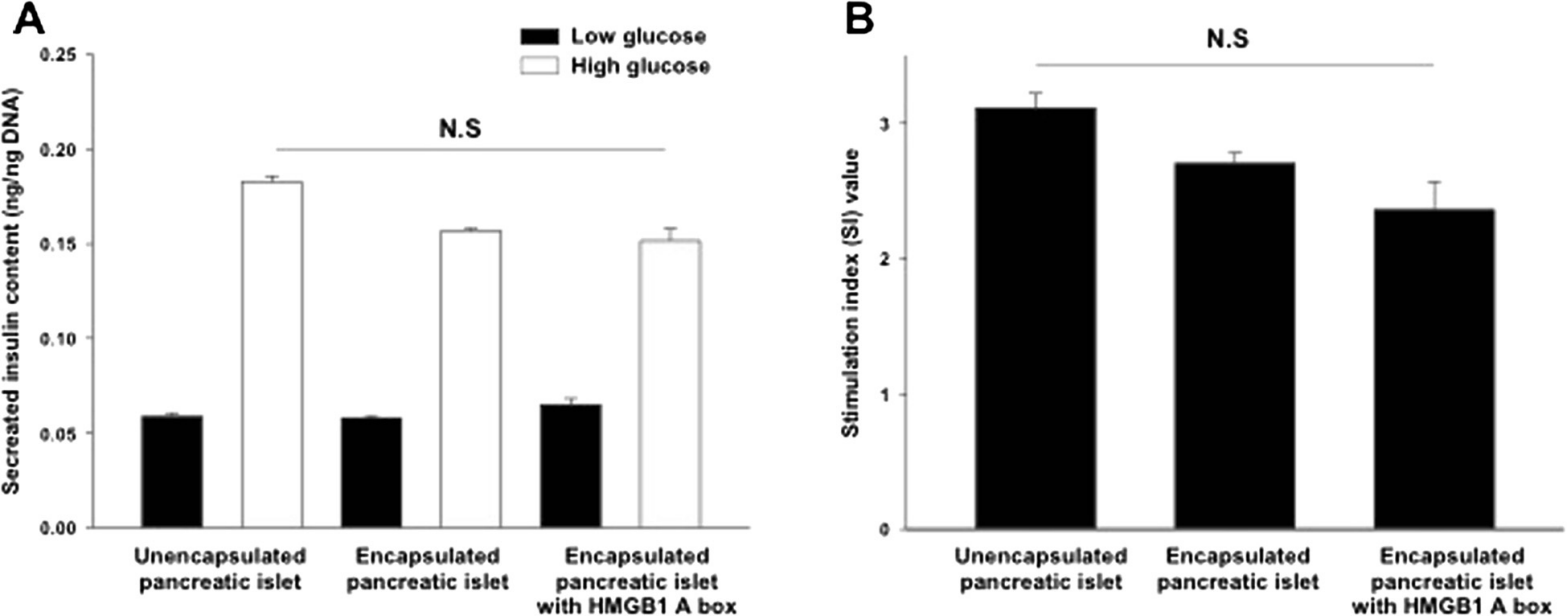
Glucose responsiveness of islets after co-encapsulation with HMGB1 A box protein. **a** Glucose-stimulated insulin secretion (GSIS) assay of the unencapsulated islet, encapsulated islet alone, and encapsulated islet with HMGB1 A box protein according to low glucose (2.8 mM, black bar) and high glucose (20.2 mM, white bar) treatment for 2 h, sequentially. **b** Stimulation index (SI) values of the unencapsulated islet, encapsulated islet alone, and encapsulated islet with HMGB1 A box protein. Data were expressed as means ± SEM (*n* = 5). N.S = no significant

It is very important to evaluate the functionality of islets in the alginate bead. Here, we carried out glucose-stimulated insulin secretion (GSIS) using low and high concentration of glucose solution (Fig. 3a). In the case unencapsulated control islets at low (2.8 mM) and high (20.2 mM) glucose concentrations, the secreted insulin content was 0.058 ± 0.002 and 0.182 ± 0.003 ng/ng DNA, respectively. With regard to the encapsulated islets, 0.058 ± 0.001 and 0.156 ± 0.001 ng/ng DNA of insulin were secreted at low and high glucose, respectively. In the encapsulated islet with HMGB1 A box protein, 0.065 ± 0.003 and 0.151 ± 0.006 ng/ng DNA of insulin were secreted at low and high glucose, respectively. There were no statistically significant differences between the groups. The stimulation index (SI value; SI = insulin content of each groups in 20.2 mM glucose solution/insulin content of each groups in 2.8 mM glucose solution) was 3.1 ± 0.1 in the unencapsulated islet, 2.7 ± 1.1 in the encapsulated islet, and 2.3 ± 0.2 in the encapsulated islet with the HMGB1 A box protein (*P* > 0.05) (Fig. 3b). In general, the SI value > 2 demonstrate that islet has good responsiveness according to different concentration of glucose. Therefore, we concluded that the functionality of the pancreatic islets in alginate bead was normally maintained even though their SI values were no significantly less than that of unencapsulated control islets.

### Co-culture of activated macrophages with islet-containing alginate bead

**Fig. 4:**
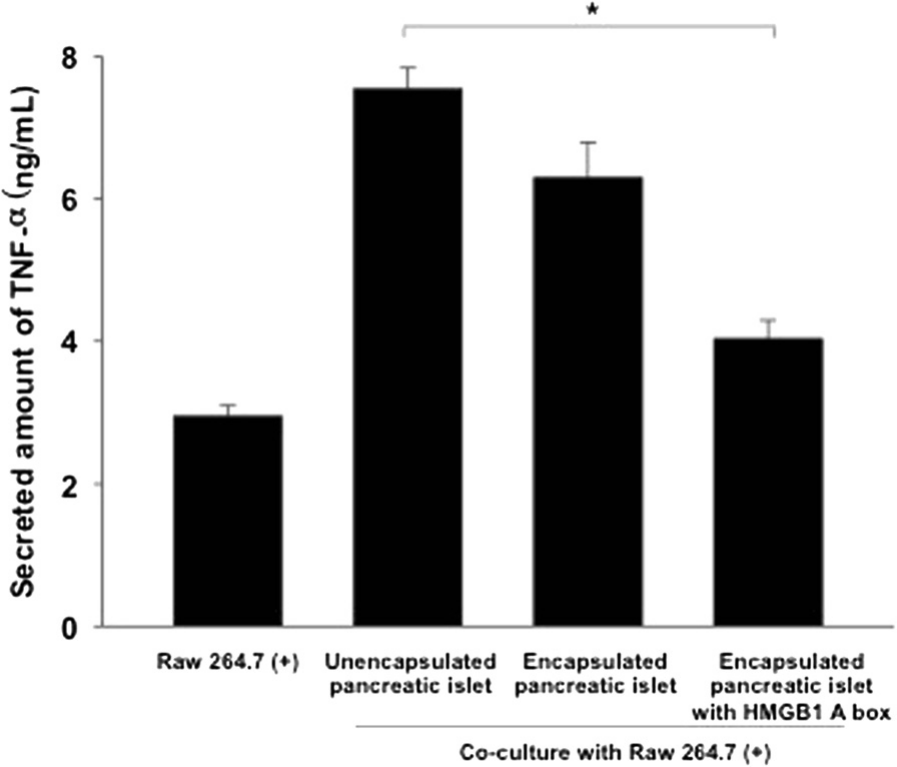
Secreted amount of TNF-α of activated macrophage during co-culture of alginate bead with islets and HMGB1 A box protein. HMGB1-activated macrophage Raw 264.7 cells were co-cultured with unencapsulated islets, encapsulated islets, and encapsulated islets with HMGB1 A box protein. Data were represented as means ± SEM (*n* = 5). *P < 0.001

Early after pancreatic islet transplantation, the activation of immune cells such as macrophages plays a crucial role in islet graft damage [16]. We tested the effect of the HMGB1 A box protein for protection of islets against macrophages (Fig. 4). Macrophage raw 264.7 cells were activated by treatment of HMGB1 protein (500 ng/ml) before co-culture with alginate beads. Without co-culture of alginate beads, the activated macrophages secreted 3.0 ± 0.1 ng/ml of TNF-α. During co-culture with unencapsulated islets, the highest TNF-α secretion level (7.5 ± 0.3 ng/ml) was significantly detected. In the case of co-culture with the encapsulated islets, the secreted amount of TNF-α was no significantly reduced (6.3 ± 0.5 ng/ml). For co-culture with the encapsulated islets with HMGB1 A box protein, the secretion of TNF-α cytokine was dramatically reduced (3.8 ± 0.3 ng/ml) (*P* < 0.001). The inhibition of the HMGB1-activated macrophage indicated that HMGB1 A box protein could be act as a competitive inhibitor against TLR and/or RAGE on macrophage. To do that, the HMGB1 A box protein could be released from the alginate bead external milieu. Therefore, the co-encapsulation of HMGB1 A box protein could be used to protect islets from the inflammation *in vivo*.

### Xenotransplantation of alginate bead with islets and HMGB1 A box protein

**Fig. 5:**
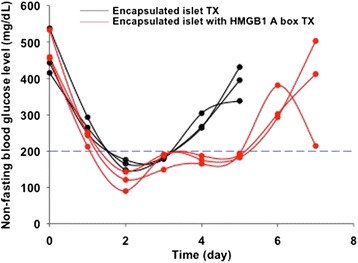
Non-fasting blood glucose levels of diabetic mice after xenotransplantation of alginate bead with rat islets and HMGB1 A box protein. Encapsulated SD-rat islets with and without HMGB1 A box protein were xenotransplanted into peritoneal cavity of diabetic BALB/c mice. Black line represented transplantation of the encapsulated islet alone, and red line represented the encapsulated islet with HMGB1 A box protein

To evaluate whether the alginate bead with islets and HMGB1 A box protein was protected *in vivo*, the alginate beads were xenotransplanted into the peritoneal cavity of diabetic mice (Fig. 5). All diabetic mice reached normal blood glucose levels (<200 mg/dL) within three days of xenotransplantation. However, this effect was not sustained in the encapsulated islet group (3 day survival). In contrast, in the encapsulated islets with HMGB1 A box protein, the mice maintained normal blood glucose levels for longer than did those without the HMGB1 A box protein (5 day survival). This effect may be secondary to longer graft survival in the HMGB1 A box protein compared to that in the encapsulated islets alone. However, the effect of HMGB1 A box protein was not dramatically stronger. The reason was that xenotransplantation had generally powerful immune reaction. So, the encapsulated islet (with and without the HMGB1 A box protein) had a weak effect in xenotransplantation models. Therefore, further studies are needed to clarify the *in vivo* effect of encapsulated islets with the HMGB1 A box protein in allotransplantation model.

## Conclusion

Islet encapsulation is one potential solution to the limitations of islet transplantation. However, encapsulation can induce the secretion of HMGB1 protein during the hypoxia-induced cell death. However, the encapsulated islet with the HMGB1 A box protein attenuated HMGB1-mediated inflammation *in vitro* and *in vivo*. However, we expected that HMGB1 A box protein might be effective in allotransplantation models.
